# Recent advances in understanding and managing cancer pain

**DOI:** 10.12688/f1000research.10817.1

**Published:** 2017-06-20

**Authors:** Marcin Chwistek

**Affiliations:** 1 Department of Hematology and Medical Oncology, Fox Chase Cancer Center/Temple Health, Philadelphia, PA, USA

**Keywords:** cancer pain, biology of cancer pain, cancer-induced bone pain, chemotherapy-induced peripheral neuropathy, opioids, cannabis, medical marijuana, neuromodulation, scrambler therapy, intrathecal analgesia, spinal cord stimulation

## Abstract

Cancer pain remains a significant clinical problem worldwide. Causes of cancer pain are multifactorial and complex and are likely to vary with an array of tumor-related and host-related factors and processes. Pathophysiology is poorly understood; however, new laboratory research points to cross-talk between cancer cells and host’s immune and neural systems as an important potential mechanism that may be broadly relevant to many cancer pain syndromes. Opioids remain the most effective pharmaceuticals used in the treatment of cancer pain. However, their role has been evolving due to emerging awareness of risks of chronic opioid therapy. Despite extensive research efforts, no new class of analgesics has been developed. However, many potential therapeutic targets that may lead to the establishment of new pharmaceuticals have been identified in recent years. It is also expected that the role of non-pharmacological modalities of treatment will grow in prominence. Specifically, neuromodulation, a rapidly expanding field, may play a major role in the treatment of neuropathic cancer pain provided that further technological progress permits the development of non-invasive and inexpensive neuromodulation techniques.

## Introduction

More than 14 million cases of cancer were diagnosed worldwide in 2012, and by 2025 the number is expected to reach more than 20 million ^1^. Despite significant advances in understanding, early detection, and treatment of cancer, progress related to the treatment of cancer pain (CP) has been slow and largely inadequate. Increased awareness of CP as a clinical problem, the development of new guidelines for treatment, and increased worldwide consumption of opioids (albeit with tremendous regional variability) have helped to reduce the burden of CP. However, the prevalence of CP remains high and continues to be one of the most feared aspects of the disease ^2, 3^. In a recent meta-analysis, based on studies published between 2005 and 2014, more than half of cancer patients receiving anti-cancer treatment and two thirds of patients with advanced and metastatic cancer report pain ^4^. Another study found that even though the quality of pharmacologic pain management has slightly improved in the last decade, 1 in 3 patients on average do not receive pain medication considered appropriate for the intensity of pain experienced ^5^.

Surprisingly, the barriers to effective pain management have remained largely the same over the last few decades. A lack of knowledge regarding pain assessment and management among clinicians is still very common. Little time is devoted to pain management in medical schools and later during postgraduate training, and misconceptions about the analgesic use and the nature of CP remain very high ^6– 8^. On the other hand, the field of oncology has been transformed. Increased survival among persons with cancer, coupled with growing complexity of the disease and introduction of new treatments, has ironically made the treatment of pain more challenging. Patients with cancer are now exposed to many therapies (some of them relatively new, such as immunotherapy) over relatively long periods of time. Many of these therapies carry a risk of considerable side effects, including pain ^9, 10^.

This article summarizes (1) recent advances in our understanding of the biology of CP, (2) emerging treatment options, (3) the evolving role of opioids, and (4) the expanding role of neuromodulation.

## New insights into the neurobiology of cancer pain: cancer pain as a distinct entity

### Is cancer pain a distinct entity?

CP is a complex biologic phenomenon that is still not well understood or classified. No specific and widely acceptable taxonomy of CP exists ^11^. In any given patient, different mechanisms can be responsible for the pain ^12, 13^. In recent years, however, new perspectives on the biology of pain caused by tumor invasion have emerged. Increasingly, such pain is understood as a result of processes that involve cross-talk between neoplastic cells and host’s immune and peripheral and central nervous systems.

In the traditional understanding of tumor growth, development of metastasis, and pain generation, the nervous system was seen primarily as a bystander. Now, it emerges as an active participant ^14^. In his influential article, Schmidt argues that CP can be seen as a harbinger of the disease ^14^. For example, benign pre-cancerous head-and-neck lesions are typically painless. However, once the lesion undergoes malignant transformation, it often becomes painful. Squamous cell cancers secrete high levels of nerve growth factor (NGF). Sequestration of NGF with an antibody has been associated with diminished pain, revealing the intricate and complex interactions between neoplastic processes and pain generation ^15, 16^.

As Brown and Ramirez state in their article, “The extent of the common pathways, signaling molecules and cell types involved in the pathological processes, resulting in both tumor development and metastasis and the generation of cancer pain, is striking” ^17^. As a result, CP is increasingly seen as a unique entity different from other pain states ^14^. Focusing on the treatment of CP and its underlying pathophysiology, argues Schmidt, may lead to a new breakthrough in not only the treatment of pain but also novel approaches to the treatment of cancer itself. However, it is important to remember that CP syndromes are a very heterogenous group and to what extent these mechanisms represent the major drivers of pain remains unknown.

### Cancer-induced bone pain

A good example of the emerging theory of CP is cancer-induced bone pain (CIBP), which now is understood as a complex pain state with nociceptive but also inflammatory and neuropathic characteristics ^11, 18^. Bone periosteum, bone marrow, and also bone matrix are highly innervated tissues that contain a network of both sensory and sympathetic neurons ^19^. Bones are primarily innervated by thinly myelinated, tropomyosin receptor kinase A-positive (TrkA ^+^) sensory nerve fibers (A-delta) and TrkA ^+^ C-fibers and receive basically no innervation through A-beta fibers. The bone nociceptors are called “silent” as they become activated only through injury or damage to the bone. Cancer cells do not destroy bone directly but rather stimulate osteoclast activation and proliferation through the promotion of the receptor activator of nuclear factor kappa-B/receptor activator of nuclear factor kappa-B ligand (RANK/RANKL) pathway. The microenvironment in the resorption “bays”, the areas between the osteoclast and bone, is highly acidic and this stimulates the sensory neurons in bone containing ion channels such as the transient receptor potential vanilloid receptor 1 (TRPV1) and acid-sensing ion channels (ASICs). Both of these channels are responsible for driving the bone pain. Additionally, several mechanosensitive ion channels are activated when sensory nerve fibers are compressed by tumor invasion. Moreover, cancer cells produce a variety of chemical mediators (prostaglandins, NGF, bradykinin, and endothelin) that can activate or sensitize bone nociceptors ^18– 21^. NGF binds to tyrosine kinase receptors on the bone nociceptors and may modulate the sensitivity and the expression of several other receptors and ion channels. Additionally, increased levels of NGF have been associated with nerve sprouting and neuroma formation within the bone. All of these mechanisms can lead to both peripheral and central sensitization. Blocking NGF has been investigated as a potential therapy for CIBP, and NGF-sequestering antibodies have shown the most promise; however, more studies are needed that will establish its safety and long-term efficacy ^22, 23^.

Traditionally, neuropathic co-analgesics have not been used widely in the treatment of CIBP ^12, 13, 24^. Given the biology of bone pain, the use of adjuvant analgesics, such as gabapentinoids, seems prudent as many patients with CIBP have neuropathic features ^25^. In animal models, gabapentin blocks the process of central sensitization most likely by modifying activation of the spinal microglia and release of pro-inflammatory cytokines, which enhance pain transmission ^26, 27^. In a small series, gabapentin has been shown to reduce breakthrough pain caused by bone metastasis ^28^ and in another study it was effective in managing postoperative orthopedic pain ^29^.

## Evolving practices in the management of cancer pain

### Pharmacological treatments

CP is a multidimensional experience that involves diverse neurophysiological changes and is also characterized by significant emotional, cognitive, and sociocultural responses. Hence, despite the development of many potential compounds in pre-clinical studies (that primarily target anti-nociception), few of them prove efficacious in clinical trials and still fewer are effective in a clinical setting. Despite all the progress made in basic research, a new class of analgesics has not been developed, and to this day, opioids remain the most effective analgesic for the treatment of CP ^30– 32^.

Opioids remain the cornerstone of pharmacological treatment of CP, but their role is evolving.

The current era of CP management began in the mid-1980s with the creation of now well-known World Health Organization (WHO) analgesic stepladder. In the decades leading up to the development of the guidelines, most patients with cancer were dying in uncontrolled pain, and the Brompton cocktail (a mixture of alcohol and cocaine) was a popular “therapeutic” remedy. The stepladder approach was a significant achievement as it provided a simple but systematic approach to the treatment of pain in patients with advanced cancer, even in developing countries ^33^. It destigmatized and legitimized the use of opioids for patients with cancer. When Jan Stjernswȁrd, the new head of the WHO’s Cancer Unit and the mastermind behind the development of the guidelines, conceived of his plan in the early 1980s, his aspiration was “to achieve world freedom from cancer pain by the year 2000” ^34^. Unfortunately, his original plan has not worked out as well as he envisioned. Today, opioids are still the cornerstone of CP treatment. However, their role in treatment has been evolving, largely due to a growing understanding of their adverse effects associated with chronic use. This is especially important in the context of longer survival in cancer and improving cure rates. Many cancer patients and cancer survivors require chronic opioid therapy (COT) (defined as greater than three months) which has been associated with increased risk of endocrinopathies, depression, sleep-disordered breathing, impaired wound healing, substance use disorders, and cognitive impairment ^9, 35^. Finding the proper balance between appropriate analgesia and minimizing the risks of associated with COT is often quite challenging. Early implementation of psychological interventions, consideration of interventional and neuromodulatory therapies, close monitoring with frequent follow-up visits, the use of naloxone for high-risk patients, and tapering of the opioid therapy whenever clinically possible are common strategies that allow for a more individualized approach to treatment ^36– 38^.

### Potential new targets in treating cancer pain

One of the new exciting areas of opioid research, promising to lead to the development of new opioid analgesics, is related to molecular discoveries of opioid action. There are three major classes of opioid receptors: mu, kappa, and delta. Mu receptors are selective for morphine, and most classic opioids exert their action by binding to these. However, the variability in response to opioids regarding both effectiveness and side effects among patients has been a well-known clinical phenomenon. Growing evidence suggests that two processes—biased agonism and alternate gene splicing—are responsible for this ^39, 40^. Exploration of both of these processes will hopefully lead to the development of new analgesics with improved tolerability.

### TRV130

The mu receptor is a member of the G protein-coupled receptor (GPCR) family. Structurally, GPCRs are seven-transmembrane domain (7TM) proteins that transmit signals into the intracellular space once an agonist binds to the receptor and activates a G-protein. However, recent discoveries indicate that GPCRs can also mediate through alternative—G protein-independent—pathways, such as β-arrestin. Therefore, drugs acting through the same opioid receptor may activate different pathways leading to different pharmacological outcomes. This process is described as biased agonism. One example of biased agonism is the action of TRV130, a novel ligand that activates G protein-biased mu receptor with little β-arrestin recruitment. It produces analgesia with no serious adverse effects and has a tolerability similar to that of morphine. It was shown to be effective in acute pain models ^41, 42^.

### IBNTxA

Opioid receptors are encoded by the *OPRM1* gene. The gene undergoes extensive alternative splicing that leads to production of the classic 7TM GPCRs but also a set of variants containing only six-transmembrane proteins ^43^. Medications, such as morphine and methadone, act through full-length 7TM mu variants. Compounds such as 3-iodobenzoyl naltrexamine ****(IBNtxA) work through 6TM. Their major benefit lies in the fact that they can mediate a potent analgesia with significantly less risk of respiratory depression and gastrointestinal or drug-liking effects. Additionally, IBNtxA is active in neuropathic and inflammatory pain models and this is quite important in CP. Truncated forms of GPCRs may provide important targets for new analgesic drug development ^44– 46^.

### Melatonin

Melatonin is a neurohormone produced in the pineal gland and exerts its action through melatonergic receptors MT1/MT2 localized in the dorsal horn of the spinal cord and in multiple areas of the central nervous system. The effect of melatonin as an anti-nociceptive agent seems to be primarily accomplished through the action on the MT2 receptor and has been shown in a number of animal models of pain perception, including neuropathic and inflammatory pain ^47^. In patients, melatonin appears to work in fibromyalgia, migraine headaches, and more importantly neuropathic pain ^48^. Agomelatine is a new class of anti-depressant that acts as MT2 receptor agonist and 5-HT 2C receptor antagonist. In pre-clinical models of neuropathic pain, the combination of agomelatine and gabapentin produced an additive effect ^49^. Agonist of MT2 receptors may represent a novel target in the treatment of neuropathic pain ^50^.

### Quetiapine

Quetiapine is an atypical anti-psychotic medication commonly used for treatment of schizophrenia and other psychiatric and neurologic disorders such as affective disorders, anxiety disorders, autism spectrum disorders, dementia, and delirium ^51^. Interestingly, it has been previously shown that quetiapine has anti-inflammatory effects and reduces joint damage and severity of arthritis in animal models. Recently, it has been evaluated as a potential analgesic in animal models of CIBP. It is hypothesized that quetiapine modulates the expression of ASICs that is increased in CIBP. These results in turn raise the possibility that TRPV and ASICs are targets for CP management ^52^.

### Cannabinoids for cancer pain

There is growing excitement among the public, patients with cancer, and researchers regarding the potential of medical marijuana for the treatment of CP ^53^. The term “medical marijuana” can be confusing. It encompasses endogenous cannabinoids, plant-derived cannabinoids—such as tetrahydrocannabinol (THC) and cannabidiol (CBD)—and synthetic cannabinoids: nabilone and dronabinol. The cannabinoid system acts as a physiologic modulator and also partakes in the organism’s homeostasis ^54^. It is involved in the pain pathways at almost every level of peripheral nerves, through the spinal cord to higher brain regions. It has also been shown to induce analgesia in pre-clinical models of both acute and chronic pain states ^55, 56^. In a recent systematic review, Whiting *et al*. concluded that there is moderate-quality evidence to support the use of cannabinoids for the treatment of chronic pain ^57^. However, the evidence of cannabinoids’ effectiveness in CP is quite limited. In a recently published meta-analysis of the use of cannabinoids for medical purposes, out of 28 included randomized trials only three were done in CP ^57– 59^. Nabiximols is an oromucosal spray of whole cannabis plant extract with a 50:50 mixture of THC and CBD (marketed under the name Sativex) and has been shown to be somewhat effective in CP; however, the studies showed a dose ceiling effect with pain ^57– 60^. In Canada, Sativex has received a Notice of Compliance with conditions for the treatment of CP unresponsive to opioids. In the US, it was granted Fast Track designation in 2014 by the US Food and Drug Administration in order to accelerate the drug’s approval for CP ^61^.

Moreover, preliminary evidence suggests that concomitant use of medical cannabis and opioids may lead to a reduction in opioid doses and opioid-related mortality. Despite currently limited evidence of the effectiveness of cannabis for the treatment of CP, it is likely that research efforts will continue to focus on examining potential ways of incorporating medical cannabis and its derivatives into CP treatment paradigms. It seems that cannabinoids can become an attractive adjuvant in the treatment of CP ^54, 62, 63^.

## Non-pharmacological treatments

### The role of neuromodulation in cancer pain management

Neuromodulation is a diverse and rapidly expanding field of medicine that may prove to be revolutionary in the treatment of neuropathic CP ^64, 65^. The modern era of neuromodulation in CP relief began in 1967 when Gol reported that repeated intracranial stimulation of the septal area resulted in effective pain control in several patients with cancer ^66^. Today, neuromodulation is described as electrical or chemical alteration of signal transmission within the nervous system by using implanted devices or—increasingly—non-invasive techniques, which results in modulation of pain signals leading to analgesia ^64, 67, 68^. It encompasses diverse therapies that range from the more widely used such as spinal cord stimulation (SCS), neuraxial drug delivery systems, and peripheral nerve stimulation (PNS) to new and less examined treatment modalities, including deep brain stimulation, repetitive transcranial magnetic stimulation (rTMS), transcranial direct current stimulation (tDCS), or motor cortex stimulation.

### Spinal cord stimulation

SCS, also known as dorsal column stimulation, is a minimally invasive, outpatient technique that involves placement of electrodes in the dorsal epidural space ^69, 70^. The electrodes are connected to a pulse generator that is implanted under the skin, typically in the buttock area. Before implantation of the device, a patient undergoes typically a week-long trial to evaluate the effectiveness of the treatment. The purported mechanism of action is based on electrical stimulation of the dorsal horn, which most likely through several mechanisms suppresses the transmission of noxious stimuli from the peripheral nerves ^64, 69, 71^. More research is needed to better define the population of cancer patients who may benefit from the therapy. However, SCS has been shown to effectively manage pain associated with chemotherapy-induced peripheral neuropathy and other neuropathic pain states ^71– 74^. A 2015 Cochrane Review concluded that existing evidence is insufficient to establish the role of SCS in treating refractory cancer-related pain. However, four case series studies, totaling 92 participants, showed diminished pain as documented by the visual analog scale and reduced analgesic use ^70^. More studies including patients with CP are needed before SCS can be widely accepted and introduced into oncological practice.

### Modified techniques of spinal cord stimulation

New modifications of the SCS have been introduced in recent years. One includes the use of high-frequency (10 kHz) stimulation that provides pain relief without the typical paresthesias experienced in the standard low-frequency SCS ^75^. Another involves dorsal root ganglion (DRG) stimulation, which has been of interest as a target of stimulation for several years. DRG contains cell bodies of primary afferent nociceptive nerve fibers, and its stimulation is thought to provide better specificity and accuracy ^76^. It is hoped that DRG stimulation may be of benefit to patients with neuropathic pain syndromes (for example, chronic postsurgical pain, which affects many patients with cancer) ^76– 79^.

### Neuraxial drug delivery systems

This form of neuromodulation involves the infusion of one or more drugs into the epidural or intrathecal (IT) space. The use of the epidural route is typically reserved for patients with a life expectancy of days to weeks, as long-term use of epidural infusion is associated with higher incidence of side effects and catheter-related problems ^80– 82^. Implantable IT pumps have been in use since 1982. However, this method remains underutilized in the treatment of intractable cancer-related pain despite a significant body of evidence documenting its effectiveness ^69, 83^. Multiple barriers, including high price, make wider utilization of IT pumps difficult. However, it seems that the lack of familiarity among oncologists regarding IT therapy as a viable, safe, and effective way of managing intractable CP and cancer patients’ reluctance to undergo an invasive procedure are the primary reasons for slow adoption of this method of treatment. There are two groups of cancer patients who seem to benefit from IT pumps the most: patients with intractable pain that failed to respond to standard therapy and patients whose narrow therapeutic window makes the use of conventional therapies unacceptable. This excellent review ^64^ discusses the use of IT pumps and SCS in great detail ^69^.

### Peripheral nerve stimulation

PNS is an exciting, rapidly evolving, and relevant field of neuromodulation today. Its current application for CP is quite limited, but as the progress in neuromodulation technology continues to advance allowing for further development of minimally invasive approaches, use of PNS methods for the treatment of cancer-related pain will likely expand. The new upcoming systems will probably use transcutaneous, easy-to-use electrodes that may revolutionize the application of PNS in the treatment of pain in general but also cancer-related pain. A non-invasive stimulation will allow PNS to be used by physicians from various backgrounds, including non-interventional specialties. The case and observational studies of the clinical use of PNS present it as an attractive modality for treatment of neuropathic pain states due to peripheral nerve injuries, nerve entrapments, or damage done to nerve plexuses. These are common complications of cancer or cancer-related therapies ^65, 84^.

### Scrambler therapy

Scrambler therapy (ST) is a novel method that, since its introduction in the early 2000s, has been used in the treatment of chronic pain, including CP ^74^. ST was discovered by Italian biophysicist Giuseppe Marineo, who describes the pain system as an “information system” and believes that chronic pain can be controlled by modulating the afferent information aspects of pain. During treatment, which involves stimulation of large and small fibers in peripheral nerves, the pathological information pain processes “are scrambled” and in effect the brain is retrained not to perceive the treated area as painful. Therefore, ST’s putative mechanism of action is thought to be different from PNS theoretical framework that is based on the gate theory of pain ^85^. There have been multiple small studies published documenting its effectiveness. However, no large clinical trials have yet been conducted ^86– 88^. The major drawbacks are its cost, inconsistent insurance coverage, and time needed to perform the treatments. Based on the published reports, the treatment seems to be operator-dependent, and a great deal of time is required to develop expertise in its application. However, based on the published studies that included more than 900 patients treated, the observed pain relief associated with ST was significant and long-lasting ^74^.

### Transcranial magnetic stimulation

Transcranial magnetic stimulation (TMS) is a non-invasive technique. It involves placement of a wire coil (connected to a stimulator that discharges a high-current pulse) over the patient’s cranium. The magnetic field produced penetrates the scalp of the patient and in turn induces the formation of electrical currents that excite or inhibit the neural tissue within the cortical and subcortical neural networks ^89, 90^. The effect of TMS depends on the position of the coil, the parameters of the stimulation (for example, high or low frequency), and its duration. TMS delivered in the form of repetitive stimulations (rTMS) was shown to produce local changes that last longer than a single stimulation. The exact mechanism of TMS for pain relief is unknown but appears to work through affecting the levels of brain-derived neurotrophic factor (BDNF). BDNF in turn has a sensitizing capacity on pain pathways and in animal models has been shown to have a maladaptive effect on descending inhibitory pain pathways ^91^. Safety is excellent and the main side effect of rTMS is a transient headache ^89, 92^. The evidence regarding the analgesic benefits of rTMS is limited ^93, 94^. A 2014 Cochrane Systematic Review concluded that single doses of high-frequency rTMS provided short-term beneficial effects in patients with chronic pain ^95^. In a recent meta-analysis, out of 21 randomized controlled trials (RCTs) evaluating the analgesic effects of rTMS in patients with neuropathic pain, 16 studies showed significant pain reduction and 5 were negative ^90^. Recent guidelines concluded that there is weak evidence of effectiveness of rTMS of M1 for the treatment of neuropathic pain and fibromyalgia ^92^. There are no RCTs examining the use of rTMS for the treatment of CP. A recent case report described successful treatment with the use of rTMS for the treatment of refractory CP in two patients in the palliative care setting ^96^. Given the non-invasive character and excellent tolerability of rTMS, using the therapy as an adjunctive treatment for CP may prove to be beneficial. However, more studies are needed to determine the exact target, parameters, and duration of such stimulation before it can be used more broadly in patients with cancer ^97^.

### Transcranial direct current stimulation

tDCS is a non-invasive, easy-to-implement, and portable technique that involves applying low-intensity (1–2 mA) current to the patient’s scalp using large sponge electrodes. The current penetrates the brain and modulates the neuronal excitability. There are two types of stimulation: anodal (stimulating) and cathodal (inhibitory). tDCS works through affecting various neurotransmitter and BDNF levels influencing the maladaptive plasticity of pain pathways ^68, 98^. Repeated sessions of anodal tDCS applied to the motor cortex contralateral to the pain side have been shown to be effective for various neuropathic pain syndromes ^99^. There are no RCTs evaluating tDCS for CP; however, in a recent pilot study, tDCS was used to treat pain associated with chemo-radiation in patients with head-and-neck cancer. tDCS of the M1 area was delivered over the course of 20 stimulation sessions during seven weeks of therapy and resulted in pain relief and attenuation of weight loss and dysphagia ^100^. The non-invasive nature of the treatment, ease of use, excellent safety profile, and low cost make tDCS a very desirable complementary technique in the treatment of CP. However, more studies defining the exact treatment protocols need to be established to allow for wide adaptation in patients with CP.

## Summary

Neurobiological discoveries indicate that CP is a separate pathophysiological entity. Complex interactions between cancer cells and the host’s neuro-immune systems are the basis of these interactions and hold promise for the development of new CP-directed therapies and possibly a new generation of non-opioid analgesics. So far, opioids do remain the most effective treatment for CP and, I believe, will continue to be the cornerstone of pharmaceutical therapies for a while. However, their use is evolving, and their role will likely diminish as potential new pharmacological and non-pharmacological treatments become more accessible and available.

Despite the overall slow progress in developing new therapies for pain, we cannot forget that the treatments we currently do have at our disposal are effective against CP in the majority of patients. In his 1942 book on pain, Charles Sherrington, a brilliant British neurophysiologist, wrote: “[pain] remains a biological enigma, a mere curse” ^101^. To our benefit, this is no longer true in a scientific sense but unfortunately remains a reality for many cancer patients around the globe ^102^. Decreasing the burden of CP depends on concerted efforts of all of us who treat persons with cancer.

## Abbreviations

7TM, seven-transmembrane domain; ASIC, acid-sensing ion channel; BDNF, brain-derived neurotrophic factor; CBD, cannabidiol; CIBP, cancer-induced bone pain; COT, chronic opioid therapy; CP, cancer pain; DRG, dorsal root ganglion; GPCR, G protein-coupled receptor; IBNtxA, 3-iodobenzoyl naltrexamine; IT, intrathecal; NGF, nerve growth factor; PNS, peripheral nerve stimulation; RCT, randomized controlled trial; rTMS, repetitive transcranial magnetic stimulation; SCS, spinal cord stimulation; ST, scrambler therapy; tDCS, transcranial direct current stimulation; THC, tetrahydrocannabinol; TMS, transcranial magnetic stimulation; TrkA ^+^, tropomyosin receptor kinase A-positive; TRPV, transient receptor potential vanilloid receptor; WHO, World Health Organization.
